# Predicting Hospitalised Paediatric Pneumonia Mortality Risk: An External Validation of RISC and mRISC, and Local Tool Development (RISC-Malawi) from Malawi

**DOI:** 10.1371/journal.pone.0168126

**Published:** 2016-12-28

**Authors:** Shubhada Hooli, Tim Colbourn, Norman Lufesi, Anthony Costello, Bejoy Nambiar, Satid Thammasitboon, Charles Makwenda, Charles Mwansambo, Eric D. McCollum, Carina King

**Affiliations:** 1 Department of Pediatrics, Section of Critical Care Medicine, Baylor College of Medicine and Texas Children’s Hospital, Houston, United States of America; 2 Department of Pediatrics, Section of Emergency Medicine, Baylor College of Medicine and Texas Children’s Hospital, Houston, United States of America; 3 Institute for Global Health, University College London, London, United Kingdom; 4 Ministry of Health, Lilongwe, Malawi; 5 Parent and Child Health Initiative, Lilongwe, Malawi; 6 Department of Pediatrics, Division of Pulmonology, Johns Hopkins School of Medicine, Baltimore, Maryland, United States of America; Liverpool School of Tropical Medicine, UNITED KINGDOM

## Abstract

**Background:**

Pneumonia is the leading infectious cause of under-5 mortality in sub-Saharan Africa. Clinical prediction tools may aide case classification, triage, and allocation of hospital resources. We performed an external validation of two published prediction tools and compared this to a locally developed tool to identify children admitted with pneumonia at increased risk for in-hospital mortality in Malawi.

**Methods:**

We retrospectively analyzed the performance of the Respiratory Index of Severity in Children (RISC) and modified RISC (mRISC) scores in a child pneumonia dataset prospectively collected during routine care at seven hospitals in Malawi between 2011–2014. RISC has both an HIV-infected and HIV-uninfected tool. A local score (RISC-Malawi) was developed using multivariable logistic regression with missing data multiply imputed using chained equations. Score performances were assessed using c-statistics, sensitivity, specificity, positive predictive value, negative predictive value, and likelihood statistics.

**Results:**

16,475 in-patient pneumonia episodes were recorded (case-fatality rate (CFR): 3.2%), 9,533 with complete data (CFR: 2.0%). The c-statistic for the RISC (HIV-uninfected) score, used to assess its ability to differentiate between children who survived to discharge and those that died, was 0.72. The RISC-Malawi score, using mid-upper arm circumference as an indicator of malnutrition severity, had a c-statistic of 0.79. We were unable to perform a comprehensive external validation of RISC (HIV-infected) and mRISC as both scores include parameters that were not routinely documented variables in our dataset.

**Conclusion:**

In our population of Malawian children with WHO-defined pneumonia, the RISC (HIV-uninfected) score identified those at high risk for in-hospital mortality. However the refinement of parameters and resultant creation of RISC-Malawi improved performance. Next steps include prospectively studying both scores to determine if incorporation into routine care delivery can have a meaningful impact on in-hospital CFRs of children with WHO-defined pneumonia.

## Introduction

Pneumonia is the number one cause of infectious under-5 child mortality in sub-Saharan Africa, attributed to 935,000 child deaths (14.9% of total) annually [1]. Malawi is a small landlocked country in southern Africa. Despite being one of the poorest countries in the world [2] it has achieved Millennium Development Goal (MDG) 4, a two-thirds reduction in under-5 child mortality [3].

In an effort to reduce child pneumonia mortality the Malawi Ministry of Health implemented the Child Lung Health Programme (CLHP) in 2000 [4]. The CLHP included the introduction of national clinical pneumonia diagnosis and management guidelines (adapted from the 2000 World Health Organization (WHO) guidelines) and a nationwide case report form for all children admitted to hospitals with pneumonia [5]. Although there has been an overall reduction in the pneumonia case fatality rate (CFR) since implementing the CLHP, minimal declines were seen in subpopulations of higher risk children with clinical danger signs and severe acute malnutrition [6]. Multiple factors may contribute to this lack of improvement, including case misclassification with resultant incorrect antibiotic usage [7], inconsistent adherence to guidelines [8], human resource constraints, medication stockouts [9] and lack of pulse oximetry and oxygen availability [10]. Therefore, one priority area could be the improved allocation of limited resources.

Clinical prediction tools may aid case classification and be used to initiate earlier escalation of care in high-risk cases, rapid in-hospital triage for resuscitation and targeted therapies or intensive care admission. Two tools have been proposed to identify hospitalized children at risk of death due to acute respiratory illness: the Respiratory Index of Severity in Children (RISC) [11] and modified Respiratory Index of Severity in Children (mRISC) [12]. RISC was developed retrospectively from a dataset collected in Soweto, South Africa from 1998–2001 in hospitalized children aged 0–24 months enrolled in a pneumococcal conjugate vaccine (PCV) randomized controlled trial, post Haemophilus influenzae type b (Hib) vaccine introduction with known HIV disease status. mRISC was developed prospectively in Western Kenya from 2009–2011 in hospitalized children aged 0–59 months post Hib vaccine and pre PCV introduction. Both studies evaluated known mortality risk factors including hypoxemia, duration of symptoms, age, and nutritional status [13–17]. To the best of our knowledge, the RISC and mRISC scores have not yet been externally validated to assess whether they are generalizable to other African pediatric inpatient populations and applicable to routine, non-clinical trial datasets that are representative of programmatic care conditions.

We aimed to externally validate the RISC and mRISC scores using routine hospital data collected prospectively through the CLHP programme during a PCV study in Malawi. We created a locally developed risk score (RISC-Malawi) that takes into account degree of hypoxemia, severity of malnutrition, level of consciousness, sex, and presence of wheezing. RISC-Malawi does not consider HIV status as this is often not available. We then compared the performance of RISC, mRISC, and RISC-Malawi.

## Methods

### Setting

Government healthcare providers collected data during routine care of children 0–59 months of age hospitalized with pneumonia at seven hospitals in Mchinji and Lilongwe districts in Malawi, between October 2011 and June 2014.

### Data collection

Clinical data was collected as part of routine care by healthcare workers using the standard CLHP inpatient case report forms [6]. Variables collected included: demographics, immunization status, past medical history, history of present illness, vital signs, anthropometrics, clinical exam findings on admission, HIV and malaria status, chest radiograph findings, and treatment course. HIV and malaria testing, while recommended, were inconsistently available [6–8].

All participating healthcare providers were trained at the beginning of the study and retrained at the study’s mid-point (early 2013) by a pediatric pulmonologist (EDM) and Malawi Ministry of Health staff. Monthly supportive supervision of healthcare providers was performed by study staff, including: direct observation of patient care, review of case classification and management guidelines, and a review of data record quality. Feedback was provided along with remediation if needed. Lay health workers, called vital sign assistants, were trained to ensure vital signs were recorded for each hospitalized patient [17]. Data entry was checked as a part of quality assurance protocols and the original paper forms were consulted as needed during data cleaning.

### Pneumonia definition

Pneumonia was defined by clinical findings as per the Malawi Ministry of Health Case Management Guidelines [6] (Table 1). Some children were receiving supplemental oxygen while their transcutaneous peripheral capillary hemoglobin oxygen saturation (SpO_2_) was obtained by study staff. Since all participating hospitals were supplied with pulse oximeters and trained in their use, we assumed these patients to have a SpO_2_ <90%. Such patients comprised less than 5% of the total study population.

**Table 1:** Malawi Ministry of Health case management guidelines for the diagnosis of pneumonia in children 0–59 months of age.

Fast breathing pneumonia(2–59 months of age)	Chest indrawing pneumonia(<2 months of age)	Chest indrawing pneumonia(2–59 months of age)	Danger sign pneumonia
Cough and/or difficulty breathing ***and***	Cough and/or difficulty breathing ***and***	Cough and/or difficulty breathing ***and***	Cough and/or difficulty breathing ***and***
Fast breathing for age^1^ ***and***	Lower chest indrawing***or***	Lower chest indrawing ***and***	At least one danger sign^2^
**No **lower chest indrawing and **no **danger signs^2^	Fast breathing for age^1^ ***and***	**No **danger signs^2^	May or may not have fast breathing for age^1^ ***or***
	**No **danger signs^2^	May or may not have fast breathing for age^1^	Lower chest indrawing

^1^≥60 breaths/minute if <2 months old, ≥50 breaths/minute 2–11 months old; ≥40 breaths/minute if 12–59 months old

^2^Danger signs include: central cyanosis; severe respiratory distress (grunting, head nodding, severe chest indrawing), stridor in a calm child, a general danger sign (inability to drink and/or breastfeed, lethargy or unconscious, convulsions), apnea (if 0–2 months of age)

### RISC and mRISC validation

We described the cases and the available RISC and mRISC variables (Table 2). Multiple variables in the mRISC tool (presence of night sweats, dehydration, and malaria test result) were not routinely documented in our dataset, with 61% missing malaria testing documentation. As such we did not externally validate mRISC. For RISC we calculated the score and performance for all age appropriate children with complete data and compared the performance of the scores against in-hospital mortality. We conducted sub-group analyses in children based on HIV status (uninfected, infected, exposed, and unknown), as well as the entire population (ALL).

**Table 2:** Criteria for RISC and mRISC Scores.

RISC (aged 0–24 months)				mRISC (aged 0–59 months)	
HIV-uninfected		HIV-infected			
SpO_2_ ≤ 90%	3 points	SpO_2_ ≤ 90%	2 points	History of loss of consciousness	1 point
**OR**		**OR**			
Chest indrawing	2 points	Chest indrawing	1 point		
Wheezing	-2 points	Wheezing	-1 point	History of unable to drink/breastfeed	1 point
Refusal to feed	1 point	Refusal to feed	1 point	History of night sweats	-1 point
WHO weight for age z-score ≤ -3	2 points	Age 0–2 months Age 3–12 months	2 points 1 point	Chest in-drawing	1 point
-2 ≤ z < -3	1 point	HIV Clinical Classification C	2 points	Not alert and awake	2 points
		HIV Clinical Classification A/B	1 point	Malaria	-1 point
				Malaria and chest in-drawing	1 point
				Dehydrated	1 point
				WHO weight for age z-score ≤ -2	1 point
Maximum score (original)	6 points	Maximum score (original)	7 points	Maximum score (original)	8 points
Maximum score (current)	6 points	Maximum score (current)	5 points	Maximum score (current)	4 points

Shaded cells indicate those variables which were available in our dataset

Score performance was assessed using sensitivity, specificity, positive predictive value (PPV), negative predictive value (NPV), and likelihood ratios (LR) for different score cut-offs. The c-statistic was used to determine how well a score discriminates between children who die during the hospital admission from those who survive to discharge. C-statistic values range from 0.5 (no discrimination) to 1.0, with a c-statistic of >0.7 considered reasonable for a clinical score performance [18].

### Local score development

Based on existing literature [6,13–17] and local clinical experience we selected the following variables *a priori* for investigation in the locally derived mortality prediction score: oxygen saturation, age-adjusted heart rate [19], malnutrition measured with mid-upper arm circumference (MUAC) and WHO weight-for-age z-score (WAZ), age, sex, presence of wheeze, unconsciousness, any danger sign, and vaccine status. Oxygen saturation, WAZ, MUAC and age were categorized as follows: SpO_2_ as severe (<90%), moderate (90–92%) and normal (≥93%); WAZ as severe (<-3 SD), moderate (-3 to -2 SD) and normal (>-2 SD); MUAC as severe (<11.5 cm), moderate (11.5–13.5 cm) and normal nutrition (>13.5 cm); age as 2–11, 12–23 and 24–59 months. Children aged <2 months were excluded as MUAC was not routinely measured in these children (14% with MUAC recorded). We did not include HIV status as a variable given that in our setting in Malawi it is inconsistently available.

The prediction score was modeled with multivariable logistic regression. Missing data were multiply imputed using chained equations [20], assuming data was missing at random. After initial data description we excluded variables based on clinical practicality and co-linearity. Goodness of fit was plotted using a risk predictiveness curve and model discrimination was assessed with the c-statistic. The patient’s log odds of mortality (derived from the model equation) was converted into their predicted probability of mortality, and a simple score derived using the methodology presented by Sullivan et al., allowing for weighting of the relative relationships to be adjusted for [21]. All analyses were done using Stata software version 14. (StataCorp. 2015. College Station, TX).

### Ethics statement

This is a secondary data analysis from a study approved by the National Health Sciences Research Committee of Malawi (protocol: 941) and the Ethics Committee of University College London (protocol: 2006/002). This analysis was submitted to the Malawi NHSRC as part of the annual ongoing project review for this study (October 2015). The Malawi NHSRC did not require retrospective consent for the analysis. The Baylor College of Medicine Institutional Review Board determined that this retrospective study does not constitute human subjects research. As data was de-identified no authorization or waiver of authorization by patients for the release of individually identifiable protected health information was required.

## Results

The dataset included 16,475 pneumonia hospitalizations in children aged 0–59 months. Of these patients 14,119 (85.7%) were aged 0–24 months of which 9,533 had complete data (67.5%) (Table 3). Children with missing data (n = 4,586) had a higher case fatality rate (CFR) than those with complete data 5.9% (5.3–6.7%) versus 2.0% (1.8–2.3%). The distribution of sex, age, and degree of malnutrition was comparable in both groups (S1 Table) and the two groups had similar proportions with danger sign, chest-indrawing, and fast breathing pneumonia.

**Table 3:** Description of inpatient pneumonia patients in dataset.

Variable		Total N (%)	0–1 months	2–23 months	24–59 months
**Oxygen saturation**	93–100%	10586 (64.3)	1166 (64.4)	7512 (64.0)	1908 (65.3)
	90–92%	1382 (8.4)	188 (10.4)	974 (8.3)	220 (7.5)
	<90%	2094 (12.7)	302 (16.7)	1533 (13.1)	259 (8.9)
	Missing	2413 (14.7)	154 (8.5)	1723 (14.7)	536 (18.3)
**WHO weight for age z-score (WAZ)**	>-2 SD	12638 (76.7)	1382 (76.4)	9157 (78.0)	2099 (71.8)
	-3 to -2 SD	1567 (9.5)	187 (10.3)	1011 (8.6)	369 (12.6)
	<-3 SD	985 (6.0)	96 (5.3)	714 (6.1)	175 (6.0)
	Missing	1285 (7.8)	145 (8.0)	860 (7.3)	280 (9.6)
**MUAC**	>13.5 cm	4557 (27.7)	11 (0.6)	3055 (26.0)	1491 (51.0)
	11.5 to 13.5cm	3382 (20.5)	34 (1.9)	2836 (24.1)	512 (17.5)
	<11.5cm	991 (6.0)	213 (11.8)	756 (6.4)	22 (0.8)
	Missing	7545 (45.8)	1552 (85.8)	5095 (43.4)	898 (30.7)
Heart rate*	Normal	2757 (16.7)	464 (25.6)	2065 (17.6)	228 (7.8)
	Low	1349 (8.2)	191 (10.6)	1077 (9.2)	81 (2.8)
	High	9863 (59.9)	990 (54.7)	6827 (58.1)	2046 (70.0)
	Missing	2506 (15.2)	165 (9.1)	1773 (15.1)	568 (19.4)
**Refusal to feed**	Yes	1430 (8.7)	211 (11.7)	1058 (9.0)	161 (5.5)
	No	11728 (71.2)	1250 (69.1)	8364 (71.2)	2114 (72.3)
	Missing	3317 (20.1)	349 (19.3)	2320 (19.8)	568 (19.4)
**Unconsciousness**	Yes	608 (3.7)	73 (4.0)	462 (3.6)	109 (3.7)
	No	12529 (76.1)	1382 (76.4)	8972 (76.4)	2175 (74.4)
	Missing	3338 (20.3)	355 (19.6)	2344 (20.0)	639 (21.9)
**Chest indrawing**	Yes	13810 (83.8)	1541 (85.1)	9931 (84.6)	2338 (80.0)
	No	1077 (6.5)	109 (6.0)	732 (6.2)	236 (8.1)
	Missing	1588 (9.6)	160 (8.8)	1079 (9.2)	349 (11.9)
**Wheeze**	Yes	4117 (25.0)	282 (15.6)	2950 (25.1)	885 (30.3)
	No	8767 (53.2)	1172 (64.8)	6225 (53.0)	1370 (46.9)
	Missing	3591 (21.8)	356 (19.7)	2567 (21.9)	668 (22.9)

*Heart rate ranges were defined per methodology presented by Fleming *et al*. with normal as: 0–3 months 133–154; 3–6 months: 129–150; 6–9 months: 123–143; 9–12 months: 118–137; 12–18 months: 112–132; 18–24 months: 106–126; 24–36 months: 100–119; 36–48 months: 94–113; 48–60 months: 89–108 [19]

### RISC (HIV-uninfected)

The RISC tool for HIV-uninfected children was applied to the total population and all the HIV-status subgroups (Table 4). There were 29 (CFR: 1.5%) and 193 (CFR: 2.0%) deaths in the HIV-uninfected and ALL patients respectively. CFR generally increased by risk-score group, with a score of 5 (only 4 patients had a maximum score of 6) having a CFR of 7.7% compared to 1.2% for 0 points in HIV-uninfected patients, and a CFR of 22.2% versus 0.7% in ALL patients (S2 Table).

**Table 4:** RISC (HIV-uninfected) performance and inpatient case fatality rate by HIV Status and Pneumonia Classification.

Classification	HIV-uninfected	HIV-infected	HIV-exposed	ALL
	(n = 1999)	(n = 152)	(n = 456)	(n = 9723)
**All Severities**				
C-statistic	0.62	0.69	0.79	0.72
(95% CI)	(0.50–0.74)	(0.55–0.84)	(0.71–0.87)	(0.68–0.76)
N	1999	152	448	9533
CFR	1.45%	6.58%	4.46%	2.02%
**Chest Indrawing**^**1**^				
C-statistic	0.63	0.48	0.85	0.70
(95% CI)	(0.35–0.91)	(0.40–0.56)	(0.72–0.97)	(0.62–0.77)
N	1044	80	248	5175
CFR	0.67%	2.50%	2.82%	0.97%
**Danger Sign**				
C-statistic	0.55	0.71	0.70	0.69
(95% CI)	(0.40–0.70)	(0.54–0.88)	(0.59–0.82)	(0.64–0.74)
N	845	65	184	3880
CFR	2.25%	12.31%	6.52%	3.45%

CI: confidence interval; N: number; CFR: in-patient case fatality rate

RISC-Malawi (MUAC) c-statistic: 0.79

^1^includes infants <2 months of age

We performed a secondary analysis to assess if the RISC (HIV-uninfected) tool had improved discrimination in subgroups of children by severity of pneumonia. Out of the 9,533 cases, 73 (0.77%) were missing pneumonia classification. The tool did not have improved discrimination of children at risk for death based on severity of pneumonia (Table 4).

At each score threshold there was no statistical difference in sensitivity, specificity, PPV, or NPV of RISC (HIV-uninfected) between the HIV-uninfected and ALL groups. We calculated the LR at score thresholds of 3 and 4 for each subgroup. With a score ≥3 (indicating high risk) the negative and positive LR was 0.53 (95% CI: 0.44–0.62) and 2.72 (95% CI: 2.35–3.00) respectively. Using this threshold, 23% of our hospitalized study population would be identified as having an increased risk of mortality. With a score ≥4 as the cut-off, the negative and positive LR was 0.74 (95% CI: 0.66–0.80) and 4.72 (95% CI: 3.83–5.90) respectively and 7% of patients would be identified as high risk (S2 Table).

### RISC (HIV-infected)

HIV clinical classification is not routinely collected as part of the medical history in Malawi. We aimed to validate a modified version of RISC (HIV-infected), by not including HIV clinical classification as a parameter. Therefore the maximum RISC (HIV-infected) score in our dataset was 5 out of the original 7. We analyzed 9,723 (68.9%) hospitalizations in the RISC (HIV-infected) validation. We calculated the inpatient case fatality rate at each score threshold, with no HIV-infected individuals scoring a maximum 5 points and in ALL patients only 51 (0.5%) scored 5 (S3 Table). In ALL patients, the CFR was 7.8% (5 points) versus 0.8% (0 points). In the ALL subgroup the tool had a c-statistic of 0.64.

### Local score development (RISC-Malawi)

A total of 14,665 patients were included in the local (RISC-Malawi) model, with 464 deaths (CFR: 3.2%). Of these 5,242 cases (35.7%) were missing at least one parameter evaluated for inclusion in the model.

Both WAZ and MUAC were investigated as indicators of malnutrition; the results of the model including MUAC is presented in Table 5. The model explained 14.7% of the variation in mortality (pseudo R^2^). Age was not included in the final models due to collinearity with MUAC and WAZ. Danger signs, vaccination status and heart rate were excluded as they did not improve model performance. The model equation is:

Log odds of in-hospital mortality = -4.67 + (moderate hypoxemia x 0.43) + (severe hypoxemia x 1.62) + (moderately malnourished x 0.55) + (severely malnourished x 1.53) + (female sex x 0.22) + (wheeze x -0.35) + (unconsciousness + 1.74)

**Table 5:** Predictors of inpatient mortality and weighted score after multiple imputation.

Predictor	Odds ratio (95% CI)	Weighted Score
**Oxygen saturation**		
Normal (≥93%)	1.00	0
Moderate hypoxemia (90–92%)^1^	1.54 (1.05, 2.28)	2
Severe hypoxemia (<90%)^2^	5.04 (4.03, 6.30)	7
**MUAC**		
Well nourished (≥13.5cm)	1.00	0
Moderately malnourished (11.5–13.5cm)^1^	1.73 (1.21, 2.48)	3
Severely malnourished (<11.5cm)^2^	4.63 (3.08, 6.97)	7
**Sex**^**1**^		
Male	1.00	0
Female	1.25 (1.02, 1.52)	1
**Wheeze present**^**1**^		
No	1.00	0
Yes	0.71 (0.53, 0.93)	-2
**Unconscious**^**2**^		
No	1.00	0
Yes	5.68 (4.01, 8.05)	8

CI: confidence interval; MUAC: mid-upper arm circumference

^1^p-value<0.05

^2^p-value<0.001

**Fig 1:**
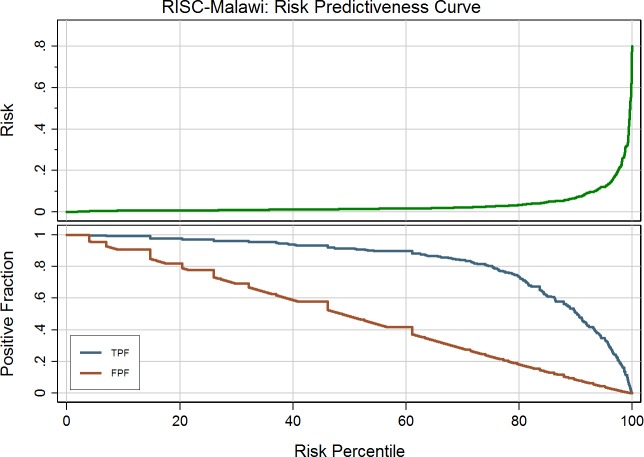
Risk Predictiveness Curve for patients aged 2–59 months old using MUAC as an indicator of nutrition status, following multiple imputation. TPF: true positive fraction; FPF: false positive fraction

The predicted risk of mortality ranged from 0–60% and was generally low, with few high-risk patients (Fig 1). The c-statistic was 0.79 (95% CI: 0.76–0.82), demonstrating a good ability to discriminate between children’s risk of mortality. The simplified score ranged from -2 to 23, with severe malnutrition and severe hypoxemia contributing 7 points each and unconsciousness contributing 8 points, as significant risks of mortality. Presence of wheeze was protective against death, as is seen with RISC. Notably, we found that a SpO_2_ of 90–92% (moderate hypoxemia) increased the odds of mortality by 1.5 times. If children with a risk score of 8 or over were classified as ‘high risk’, this would give a sensitivity of 57% and specificity of 88%, and would result in 10% of patients being selected. However, taking a more moderate score threshold of 5 gives a sensitivity and specificity of 82% and 73% respectively, and results in 25% of patients classified as high risk (Table 6).

**Table 6:** RISC-Malawi (MUAC) sensitivity, specificity, and percentage of hospitalized patients identified as high risk at different score thresholds.

Score	High Risk	Sensitivity	Specificity	LR+	LR-
3	50%	92%	44%	1.64	0.18
5	25%	82%	73%	3.04	0.25
6	22%	75%	79%	3.57	0.32
7	17%	67%	84%	4.19	0.39
8	10%	57%	88%	4.75	0.49
15	1%	12%	99%	12.00	0.89

LR+/-: Positive and Negative Likelihood Ratio

Using WAZ instead of MUAC as an indicator of nutritional status, predicted risk ranged from 0–67% with a pseudo-R^2^ of 16.4% and c-statistic of 0.80 (95% CI: 0.77–0.83) (S3 Table). Classifying the top 10% of patients as high risk with a score cut-off of 7 would give a sensitivity of 52% and specificity of 88%.

## Discussion

We investigated the use of RISC and mRISC, alongside a locally developed RISC-Malawi score, utilizing an existing prospectively collected dataset. Given that the majority of the parameters used in mRISC were not part of the routine CLHP protocol we were unable to externally validate this tool. The RISC tool includes two separate scoring systems for HIV-uninfected and infected populations. As our data set did not include HIV Clinical Classification it was not possible to appropriately evaluate the RISC (HIV-infected) score. The RISC (HIV-uninfected) score performed well with a c-statistic of 0.72 in the ALL group, and as high as 0.79 in the HIV-Exposed group. RISC-Malawi, unlike RISC, does not include HIV status as a parameter. It performed similarly with a c-statistic of 0.79 and 0.80, when considering either MUAC or WAZ as the measure of nutritional status, with hypoxemia, malnutrition and unconsciousness significant predictors of mortality.

A key feature of the RISC score is its stratification by HIV status. In our population, HIV status was missing in 73% of cases, something that is unfortunately common for children in many HIV-endemic clinical settings with weak pediatric HIV testing programs [22]. Despite this, performance was reasonable, although expectedly lower than that seen in the original study, with 95% confidence intervals of the c-statistics for all HIV status subgroups in the range of the original RISC study (0.92, 95% CI: 0.74–0.93) (S4 Table). Clinical prediction tools often perform best in the population in which they were developed and there may be other factors contributing to mortality in our study population that were not incorporated into the score (e.g. malaria co-infection). As there is collinearity between malnutrition and HIV status, it may also be the case that the effect of HIV is being accounted for by the inclusion of WAZ in the RISC score.

There has been little reduction, despite multiple interventions, in the CFRs of fast-breathing pneumonia in children complicated by severe acute malnutrition and children with danger-sign pneumonia in Malawi [6]. We performed a sensitivity analysis to determine if RISC (HIV-uninfected) was better at identifying high-risk children amongst those diagnosed with danger-sign pneumonia, however it did not have improved discrimination when applied to this subset. Given the higher CFR in cases with danger-signs or severe acute malnutrition, continued research efforts are needed to better identify subsets of children within these high-risk groups that may have reversible conditions amenable to appropriate expedited care [23,24].

Determining a cut-off score to guide clinical management is challenging, with the need to balance sensitivity and specificity. For RISC (HIV-uninfected) we calculated classification performance measures at thresholds of 3 and 4. Using a score of 3 (sensitivity 59%, specificity 78%) has a reasonable balance, although results in classifying 23% of patients as high risk. With a score threshold of 4 the sensitivity was 32.6%, specificity of 93.1% with 7% of the data set was identified as high risk. Whether this could be useful for identifying children at higher risk of pneumonia mortality in practice would depend on the specific resources and referrals available for that setting. In comparison with the original RISC study, the sensitivity of RISC (HIV-uninfected) was much lower at each score threshold in our population (S4 Table). This difference may, in part, reflect the overall lower CFR in our dataset vs the original RISC study (3.2% vs 7.2%).

We were limited in our ability to fully assess the RISC (HIV-infected) score, as we did not have records of the HIV clinical classification for participants. Based on our clinical experience in Malawi, this is not often discussed with patients, especially in acute care settings, making it difficult for them to recall in subsequent health encounters. Including such a parameter limits the practicality of the tool. A notable limitation to the RISC (HIV-infected) score is that clinical practice for the management of HIV exposure and infection in infants is very different in Malawi than it was in Soweto, South Africa in 1998 when RISC was developed [25–27]. The RISC (HIV-infected) score was developed in a population that, unlike Malawi in 2011, did not have routine access to antiretroviral therapy or compulsory usage of co-trimoxazole for *Pneumocystis jirovecii* Pneumonia (PJP formerly PCP) prophylaxis [28,29]. Both interventions could have a significant impact on the prevalence and mortality rate of pneumonia in HIV-infected children and therefore the performance of a mortality prediction tool.

Our locally developed score, RISC-Malawi, performed well, however there were high levels of missing data for MUAC in our dataset (40%). Using WAZ as an alternative measure of nutritional status with only 8% missing data had equal, if not better, model performance. However, the practicality of having a health worker calculate a child’s weight-for-age z-score would hinder the tool’s operationalization–also a limitation of RISC (HIV-uninfected) and mRISC. A potential solution could be the implementation of an mHealth tool that supports diagnostic and treatment decisions in which the calculations could be done on behalf of the health care worker. Notably, we found that a moderate range of hypoxemia (SpO_2_ 90–92%) is associated with increased mortality risk. This finding is likely to have important implications for determining what oxygen saturation threshold is most appropriate for hospital referral of outpatients with respiratory disease as well as whether the current <90% threshold for initiating in-hospital oxygen treatment is sufficient. Further research is needed.

We presented the sensitivity and specificity for RISC-Malawi based on classifying the top 10% versus 25% of children as high risk. The score threshold for defining ‘high risk’ is not just a case of optimizing sensitivity versus specificity, but also includes the resources available, local context and potential interventions. In our case, considering a score of 5 improves sensitivity compared to a score of 8 (82% vs. 57%), but results in 25% of children being identified for further treatment instead of 10%. Depending on local resources, it may not be possible to have a lower risk threshold, and context specific factors are needed to define the threshold that is feasible but effective. Real-life application in clinical settings is required to find the optimal score cut-off.

There were multiple limitations to our study, including high levels of missing data and the fact that children with missing data had a higher case fatality rate. We do not have a clear explanation for this difference. One may speculate that it may be more difficult to collect accurate information from children who present in critical condition. Alternatively, there may have been retrospective death reporting, and hence incomplete data collection in some cases. Post-discharge mortality in children admitted with pneumonia may be greater than in-hospital mortality, which we could not take into account [30]. As this data was collected at multiple sites there may have been individual and clinical practice variances that could have contributed to mortality, as well as variance in data quality and completeness [8]. HIV and malaria testing were not documented in a large proportion of our dataset. This is attributed to a variety of factors including clinician variability, inadequate staffing, and test kit stockouts. Clinical findings associated with malaria and pneumonia overlap making it challenging to correctly distinguish between these diseases without diagnostic tests [31].

We have shown that the RISC (HIV-uninfected) score is a valid prediction tool to identify Malawian children aged 0–24 months with pneumonia at increased risk of in-hospital mortality. Our new tool, RISC-Malawi, for usage in children aged 2–59 months has similar calibration to that of RISC (HIV-uninfected). However, RISC-Malawi has fewer parameters, can use MUAC as a marker of malnutrition instead of WAZ, and can be used in a wider age range of children. Unlike RISC (HIV-uninfected), RISC-Malawi reflects disease epidemiology after the introduction of pneumococcal conjugate, Haemophilus influenzae type b, and rotaviral vaccines as well as antiretroviral and co-trimoxazole prophylaxis. These factors may make RISC-Malawi more readily operationalized and representative. It remains to be determined if either tool could be useful in a clinical context or in health services research as neither has been prospectively applied and studied. Future research should include the incorporation of these tools into existing hospital case identification and management guidelines, including mHealth applications, to determine the utility of such tools. Evaluation of the performance of RISC (HIV-uninfected) and RISC-Malawi in outpatient children with pneumonia is also needed to determine if the scores are relevant outside of hospitals. Selection of the most appropriate tool will depend on local practices, namely HIV diagnosis and management procedures, method of malnutrition assessment, oxygen saturation measurements and malaria testing.

## Supporting Information

S1 Table Comparison of subjects with missing data (excluded from RISC (HIV-Uninfected) analysis) and with complete data. (PDF)

S2 Table Case Fatality Rate (CFR) with 95% Confidence Intervals (CI), Positive Predictive Value (PPV), and Likelihood Ratios (LR) at RISC (HIV-Uninfected) cutoffs by group. (PDF)

S3 Table Predictors of in-patient mortality and weighted score after multiple imputation, using WAZ. (PDF)

S4 Table Comparison of the Sensitivity and Specificity of RISC (HIV Uninfected) in our Malawian study population (ALL) with that of the South African RISC (RISC-SA) study. (PDF)

## References

[1] Liu L, Oza S, Hogan D, Perin J, Rudan I, Lawn JE, et al Global, regional, and national causes of child mortality in 2000–13, with projections to inform post-2015 priorities: an updated systematic analysis. The Lancet. 2015;385: 430–440. 10.1016/S0140-6736(14)61698-6 25280870

[2] The World Bank. GDP per capita (current US$) | Data | Table [Internet]. [cited 24 Feb 2016]. Available: http://data.worldbank.org/indicator/NY.GDP.PCAP.CD?order=wbapi_data_value_2013+wbapi_data_value&sort=asc

[3] Kanyuka M, Ndawala J, Mleme T, Chisesa L, Makwemba M, Amouzou A, et al Malawi and Millennium Development Goal 4: a Countdown to 2015 country case study. Lancet Glob Health. 2016;4: e201–e214. 10.1016/S2214-109X(15)00294-6 26805586

[4] Enarson PM, Gie RP, Mwansambo CC, Maganga ER, Lombard CJ, Enarson DA, et al Reducing Deaths from Severe Pneumonia in Children in Malawi by Improving Delivery of Pneumonia Case Management. Ferrand RA, editor. PLoS ONE. 2014;9: e102955 10.1371/journal.pone.0102955 25050894 PMC4106861

[5] Enarson PM, Gie R, Enarson DA, Mwansambo C. Development and Implementation of a National Programme for the Management of Severe and Very Severe Pneumonia in Children in Malawi. PLoS Med. 2009;6: e1000137 10.1371/journal.pmed.1000137 19901978 PMC2766047

[6] Lazzerini M, Seward N, Lufesi N, Banda R, Sinyeka S, Masache G, et al Mortality and its risk factors in Malawian children admitted to hospital with clinical pneumonia, 2001–12: a retrospective observational study. Lancet Glob Health. 2016;4: e57–e68. 10.1016/S2214-109X(15)00215-6 26718810 PMC5495601

[7] Enarson PM, Gie RP, Mwansambo CC, Chalira AE, Lufesi NN, Maganga ER, et al Potentially Modifiable Factors Associated with Death of Infants and Children with Severe Pneumonia Routinely Managed in District Hospitals in Malawi. Faragher EB, editor. PLOS ONE. 2015;10: e0133365 10.1371/journal.pone.0133365 26237222 PMC4523211

[8] Bjornstad E, Preidis GA, Lufesi N, Olson D, Kamthunzi P, Hosseinipour MC, et al Determining the quality of IMCI pneumonia care in Malawian children. Paediatr Int Child Health. 2014;34: 29–36. 10.1179/2046905513Y.0000000070 24091151 PMC4424282

[9] Lufesi NN, Andrew M, Aursnes I. Deficient supplies of drugs for life threatening diseases in an African community. BMC Health Serv Res. 2007;7: 86 10.1186/1472-6963-7-86 17573958 PMC1906855

[10] McCollum ED, Bjornstad E, Preidis GA, Hosseinipour MC, Lufesi N. Multicenter study of hypoxemia prevalence and quality of oxygen treatment for hospitalized Malawian children. Trans R Soc Trop Med Hyg. 2013;107: 285–292. 10.1093/trstmh/trt017 23584373 PMC4030433

[11] Reed C, Madhi SA, Klugman KP, Kuwanda L, Ortiz JR, Finelli L, et al Development of the Respiratory Index of Severity in Children (RISC) Score among Young Children with Respiratory Infections in South Africa. Jhaveri R, editor. PLoS ONE. 2012;7: e27793 10.1371/journal.pone.0027793 22238570 PMC3251620

[12] Emukule GO, McMorrow M, Ulloa C, Khagayi S, Njuguna HN, Burton D, et al Predicting Mortality among Hospitalized Children with Respiratory Illness in Western Kenya, 2009–2012. Metcalfe JZ, editor. PLoS ONE. 2014;9: e92968 10.1371/journal.pone.0092968 24667695 PMC3965502

[13] Sehgal V, Sethi GR, Sachdev HP, Satyanarayana L. Predictors of mortality in subjects hospitalized with acute lower respiratory tract infections. Indian Pediatr. 1997;34: 213–219. 9282488

[14] Tiewsoh K, Lodha R, Pandey RM, Broor S, Kalaivani M, Kabra SK. Factors determining the outcome of children hospitalized with severe pneumonia. BMC Pediatr. 2009;9: 15 10.1186/1471-2431-9-15 19236689 PMC2651138

[15] Djelantik IGG. Case Fatality Proportions and Predictive Factors for Mortality among Children Hospitalized with Severe Pneumonia in a Rural Developing Country Setting. J Trop Pediatr. 2003;49: 327–332. 14725409 10.1093/tropej/49.6.327

[16] Lupisan SP, Ruutu P, Erma Abucejo-Ladesma P, Quiambao BP, Gozum L, Sombrero LT, et al Predictors of death from severe pneumonia among children 2–59 months old hospitalized in Bohol, Philippines: implications for referral criteria at a first-level health facility. Trop Med Int Health TM IH. 2007;12: 962–971. 10.1111/j.1365-3156.2007.01872.x 17697091

[17] Olson D, Preidis GA, Milazi R, Spinler JK, Lufesi N, Mwansambo C, et al Task shifting an inpatient triage, assessment and treatment programme improves the quality of care for hospitalised Malawian children. Trop Med Int Health TM IH. 2013;18: 879–886. 10.1111/tmi.12114 23600592 PMC3683117

[18] Hosmer David W., Lemeshow Stanley. Applied Logistic Regression. 2nd ed. New York, NY: John Wiley & Sons; 2000.

[19] Fleming S, Thompson M, Stevens R, Heneghan C, Plüddemann A, Maconochie I, et al Normal ranges of heart rate and respiratory rate in children from birth to 18 years of age: a systematic review of observational studies. Lancet Lond Engl. 2011;377: 1011–1018. 10.1016/S0140-6736(10)62226-X PMC3789232 21411136

[20] White IR, Royston P, Wood AM. Multiple imputation using chained equations: Issues and guidance for practice. Stat Med. 2011;30: 377–399. 10.1002/sim.4067 21225900

[21] Sullivan LM, Massaro JM, D’Agostino RB. Presentation of multivariate data for clinical use: The Framingham Study risk score functions. Stat Med. 2004;23: 1631–1660. 10.1002/sim.1742 15122742

[22] Kelly MS, Wirth KE, Steenhoff AP, Cunningham CK, Arscott-Mills T, Boiditswe SC, et al Treatment Failures and Excess Mortality Among HIV-Exposed, Uninfected Children With Pneumonia. J Pediatr Infect Dis Soc. 2015;4: e117–e126. 10.1093/jpids/piu092 PMC4681380 26582879

[23] Ramachandran P, Nedunchelian K, Vengatesan A, Suresh S. Risk factors for mortality in community acquired pneumonia among children aged 1–59 months admitted in a referral hospital. Indian Pediatr. 2012;49: 889–895. 22791667 10.1007/s13312-012-0221-3

[24] Chisti MJ, Tebruegge M, La Vincente S, Graham SM, Duke T. Pneumonia in severely malnourished children in developing countries–mortality risk, aetiology and validity of WHO clinical signs: a systematic review. Trop Med Int Health. 2009;14: 1173–1189. 10.1111/j.1365-3156.2009.02364.x 19772545

[25] Impact of an Innovative Approach to Prevent Mother-to-Child Transmission of HIV—Malawi, July 2011–September 2012 [Internet]. [cited 26 Feb 2016]. Available: http://www.cdc.gov/mmwr/preview/mmwrhtml/mm6208a3.htm PMC4604864 23446514

[26] Sinunu MA, Schouten EJ, Wadonda-Kabondo N, Kajawo E, Eliya M, Moyo K, et al Evaluating the Impact of Prevention of Mother-to-Child Transmission of HIV in Malawi through Immunization Clinic-Based Surveillance. Braitstein P, editor. PLoS ONE. 2014;9: e100741 10.1371/journal.pone.0100741 24968298 PMC4072708

[27] Clinical Management of HIV in Children and Adults—Malawi-HIV-Guidelines-2014.pdf [Internet]. [cited 26 Feb 2016]. Available: http://www.emtct-iatt.org/wp-content/uploads/2015/09/Malawi-HIV-Guidelines-2014.pdf

[28] Meyers T, Dramowski A, Schneider H, Gardiner N, Kuhn L, Moore D. Changes in Pediatric HIV-Related Hospital Admissions and Mortality in Soweto, South Africa, 1996–2011: Light at the End of the Tunnel? JAIDS J Acquir Immune Defic Syndr. 2012;60: 503–510. 10.1097/QAI.0b013e318256b4f8 22487588 PMC3404242

[29] Preidis GA, McCollum ED, Mwansambo C, Kazembe PN, Schutze GE, Kline MW. Pneumonia and Malnutrition are Highly Predictive of Mortality among African Children Hospitalized with Human Immunodeficiency Virus Infection or Exposure in the Era of Antiretroviral Therapy. J Pediatr. 2011;159: 484–489. 10.1016/j.jpeds.2011.02.033 21489553 PMC4423795

[30] Wiens MO, Pawluk S, Kissoon N, Kumbakumba E, Ansermino JM, Singer J, et al Pediatric Post-Discharge Mortality in Resource Poor Countries: A Systematic Review. Bonkowsky JL, editor. PLoS ONE. 2013;8: e66698 10.1371/journal.pone.0066698 23825556 PMC3692523

[31] Bassat Q, Machevo S, O’Callaghan-Gordo C, Sigaúque B, Morais L, Díez-Padrisa N, et al Distinguishing malaria from severe pneumonia among hospitalized children who fulfilled integrated management of childhood illness criteria for both diseases: a hospital-based study in Mozambique. Am J Trop Med Hyg. 2011;85: 626–634. 10.4269/ajtmh.2011.11-0223 21976562 PMC3183767

