# Correction: Predicting Hospitalised Paediatric Pneumonia Mortality Risk: An External Validation of RISC and mRISC, and Local Tool Development (RISC-Malawi) from Malawi

**DOI:** 10.1371/journal.pone.0193557

**Published:** 2018-02-22

**Authors:** Shubhada Hooli, Tim Colbourn, Norman Lufesi, Anthony Costello, Bejoy Nambiar, Satid Thammasitboon, Charles Makwenda, Charles Mwansambo, Eric D. McCollum, Carina King

In the Local score development (RISC-Malawi) subsection of the Results section, there is an error in the model equation following the second paragraph. The correct equation is:

Log odds of in-hospital mortality = -4.67 + (moderate hypoxemia x 0.43) + (severe hypoxemia x 1.62) + (moderately malnourished x 0.55) + (severely malnourished x 1.53) + (female sex x 0.22) + (wheeze x -0.35) + (unconsciousness x 1.74)
