# Supplementary material for: Predicting Hospitalised Paediatric Pneumonia Mortality Risk: An External Validation of RISC and mRISC, and Local Tool Development (RISC-Malawi) from Malawi
Source: PLoS One. 2016 Dec 28;11(12):e0168126. doi: 10.1371/journal.pone.0168126 (PMC5193399; doi:10.1371/journal.pone.0168126)
Supplement: S1 Table — (PDF) [file pone.0168126.s001.pdf]

S1 Table: Comparison of subjects with missing data (excluded from RISC (HIV-uninfected) analysis) and with complete data

Variable	Complete Data n=9533	Missing Data[1] n=4586
Overall CFR	2.0% (1.8-2.3%)	5.9% (5.3-6.7%)
Sex	Male 56.3% Female 43.7%	Male 55.7% Female 44.3%
Age	8.7 months	9.4 months
Moderate malnutrition[2]	9.8%	9.2%
Severe malnutrition[2]	6.12%	7.5%
Pneumonia severity		
Danger Sign	n=3880 CFR 3.5% (2.9-4.1%)	n=920 CFR 13.5% (11.3-15.9%)
Chest-indrawing	n=5175 CFR 1.0% (0.7-1.3%)	n=2640 CFR 4.6% (3.8-5.5%)
Fast breathing	n=365 CFR 0.5% (0.1-2.0%)	n=483 CFR 1.7% (0.7-3.2%)

Malnutrition defined by WHO weight-for-age z-score criteria
CFR: Case Fatality Rate
[1]Subjects with missing data were excluded from the RISC (HIV-uninfected) external validation.
[2]Missing in 22% of missing data cohort
