# Supplementary material for: Predicting Hospitalised Paediatric Pneumonia Mortality Risk: An External Validation of RISC and mRISC, and Local Tool Development (RISC-Malawi) from Malawi
Source: PLoS One. 2016 Dec 28;11(12):e0168126. doi: 10.1371/journal.pone.0168126 (PMC5193399; doi:10.1371/journal.pone.0168126)
Supplement: S2 Table — (PDF) [file pone.0168126.s002.pdf]

S2 Table: RISC (HIV-uninfected) c-statistics, likelihood ratios (LR), and case fatality rates (CFR) at score cutoffs by subgroup

C-statistic	**HIV-uninfected n=1999 CFR 1.45%** 0.62 (0.5-0.74)	**HIV-unknown n=6934 CFR 1.93%** 0.72 (0.67-0.77)	**HIV-infected n=152 CFR 6.58%** 0.69 (0.55-0.84)	**HIV-exposed n=448 CFR 4.46%** 0.79 (0.71-0.87	**ALL n=9533 CFR 2.02%** 0.72 (0.68-0.76)
**≤ 3**	1868 (93.45%)	6453 (93.06%)	130 (85.53%)	378 (84.38%)	8829 (92.62%)
**CFR (95%CI)**	1.07% (0.66-1.65%)	1.44% (1.16-1.76%)	5.38% (2.19-10.78%)	2.65% (1.28-4.81%)	1.47% (1.23-1.75%)
**≥ 4**	131 (6.55%)	481 (6.94%)	22 (14.47%)	70 (15.62%)	704 (7.38%)
**CFR (95%CI)**	6.87% (3.19-12.64%)	8.52% (6.19-11.39%)	13.64% (2.91-34.9%)	14.29% (7.07-24.71%)	8.95% (6.94-11.30%)
**LR+|LR-**	5.01|0.74	4.73|0.74	2.24|0.81	3.57|0.58	4.76|0.72
**≤ 2**	1590 (79.54%)	5360 (77.30%)	104 (68.42%)	289 (64.51%)	7343 (77.03%)
**CFR (95%CI)**	1.01% (0.58-1.63%)	1.01% (0.76-1.31%)	4.81% (1.58-10.86%)	1.38% (0.38-3.50%)	1.08% (0.85-1.33%)
**≥ 3**	409 (20.46%)	1574 (22.70%)	48 (31.58%)	159 (35.49%)	2190 (22.97%)
**CFR (95%CI)**	3.18% (1.70-5.37%)	5.08% (4.05-6.29%)	10.42% (3.47-22.66%)	10.06% (5.86-15.83%)	5.21% (4.31-6.22%)
**LR+|LR-**	2.23|0.69	2.72|0.52	1.65|0.72	2.39|0.30	2.66|0.53

LR+: Positive Likelihood Ratio
LR-: Negative Likelihood Ratio
