# Supplementary material for: Predicting Hospitalised Paediatric Pneumonia Mortality Risk: An External Validation of RISC and mRISC, and Local Tool Development (RISC-Malawi) from Malawi
Source: PLoS One. 2016 Dec 28;11(12):e0168126. doi: 10.1371/journal.pone.0168126 (PMC5193399; doi:10.1371/journal.pone.0168126)
Supplement: S3 Table — (PDF) [file pone.0168126.s003.pdf]

S3 Table: Predictors of in-patient mortality and weighted score after multiple imputation, using WHO WAZ

Predictor	Odds ratio (95% CI)	Weighted Score
**Oxygen saturation**		
**Normal (≥93%)**	1.00	0
**Moderate hypoxemia (90-92%)**[1]	1.49 (1.02, 2.20)	1
**Severe hypoxemia (<90%)**[2]	4.40 (3.48, 5.57)	5
WAZ		
**Well nourished (≥-2 SD)**	1.00	0
**Moderately malnourished (≥-3 - <-2 SD)**[2]	2.88 (2.11, 3.93)	3
**Severely malnourished (<-3 SD)**[2]	6.04 (4.53, 8.06)	6
**Sex**[2]		
**Male**	1.00	0
**Female**	1.38 (1.13, 1.68)	1
**Wheeze present**[1]		
**No**	1.00	0
**Yes**	0.71 (0.54, 0.94)	-1
**Unconscious**[2]		
**No**	1.00	0
**Yes**	5.17 (3.71, 7.23)	5

CI: confidence interval; WAZ: weight-for-age z-score

[1]p-value<0.05; [2]p-value<0.001
