# Supplementary material for: Predicting Hospitalised Paediatric Pneumonia Mortality Risk: An External Validation of RISC and mRISC, and Local Tool Development (RISC-Malawi) from Malawi
Source: PLoS One. 2016 Dec 28;11(12):e0168126. doi: 10.1371/journal.pone.0168126 (PMC5193399; doi:10.1371/journal.pone.0168126)
Supplement: S4 Table — (PDF) [file pone.0168126.s004.pdf]

S4 Table: Comparison of the sensitivity and specificity of RISC (HIV-uninfected) in our Malawian study population (ALL) with that of the South African RISC (RISC-SA) study population

RISC Score	Sensitivity ALL	Sensitivity RISC-SA	Specificity ALL	Specificity RISC-SA
**0**	100%	100%	0%	0%
**1**	91%	100%	26%	48%
**2**	85%	100%	33%	67%
**3**	59%	94%	78%	78%
**4**	33%	72%	93%	90%
**5**	11%	47%	99%	96%
**6**	2%	16%	100%	99%
